# *PSEN1 c.1292C*<*A* Variant and Early-Onset Alzheimer’s Disease: A Scoping Review

**DOI:** 10.3389/fnagi.2022.860529

**Published:** 2022-07-22

**Authors:** Maribel Orozco-Barajas, Yulisa Oropeza-Ruvalcaba, Alejandro A. Canales-Aguirre, Victor J. Sánchez-González

**Affiliations:** ^1^Doctorado en Biociencias, Centro Universitario de los Altos, Universidad de Guadalajara, Guadalajara, Mexico; ^2^Centro de Atención Psicológica, Tepatitlán de Morelos, Mexico; ^3^Departamento de Biotecnología Médica y Farmacéutica, Centro de Investigación y Asistencia en Tecnología y Diseño del Estado de Jalisco A. C. (CIATEJ), Guadalajara, Mexico; ^4^Departamento de Clínicas, Centro Universitario de los Altos, Universidad de Guadalajara, Guadalajara, Mexico

**Keywords:** A431E, *c.1292C<A*, *PSEN1*, EOAD, founder effect, dementia, ADAD, scoping review

## Abstract

Alzheimer’s disease (AD) is the most common cause of dementia, characterized by progressive loss of cognitive function, with β-amyloid plaques and neurofibrillary tangles being its major pathological findings. Although the disease mainly affects the elderly, c. 5–10% of the cases are due to *PSEN1*, *PSEN2*, and *APP* mutations, principally associated with an early onset of the disease. The A413E (rs63750083) *PSEN1* variant, identified in 2001, is associated with early-onset Alzheimer’s disease (EOAD). Although there is scant knowledge about the disease’s clinical manifestations and particular features, significant clinical heterogeneity was reported, with a high incidence of spastic paraparesis (SP), language impairments, and psychiatric and motor manifestations. This scoping review aims to synthesize findings related to the A431E variant of *PSEN1.* In the search, we followed the Preferred Reporting Items for Systematic Reviews and Meta-Analyses (PRISMA) statement and the guidelines proposed by Arksey and O’Malley. We searched and identified 247 studies including the A431E variant of *PSEN1* from 2001 to 2021 in five databases and one search engine. After the removal of duplicates, and apply inclusion criteria, 42 studies were finally included. We considered a narrative synthesis with a qualitative approach for the analysis of the data. Given the study sample conformation, we divided the results into those carried out only with participants carrying A431E (seven studies), subjects with *PSEN* variants (11 studies), and variants associated with EOAD in *PSEN1*, *PSEN2*, and *APP* (24 studies). The resulting synthesis indicates most studies involve Mexican and Mexican-American participants in preclinical stages. The articles analyzed included carrier characteristics in categories such as genetics, clinical, imaging techniques, neuropsychology, neuropathology, and biomarkers. Some studies also considered family members’ beliefs and caregivers’ experiences. Heterogeneity in both the studies found and carrier samples of EOAD-related gene variants does not allow for the generalization of the findings. Future research should focus on reporting data on the progression of carrier characteristics through time and reporting results independently or comparing them across variants.

## Introduction

Alzheimer’s disease causes c. 70% of dementia cases (World Health Organization, 2020), which is a neurodegenerative disease resulting in progressive cognitive deficits due to plaques and tangles accumulation leading to inflammation and oxidative stress responses (Amponsah et al., 2021).

Most cases are related to susceptibility genes and risk factors such as age, obesity, hypertension, diabetes, depression, and others (Livingston et al., 2020). Variants in the causality genes, including *PSEN1, PSEN2*, and *APP* (identified with high penetrance), are responsible for only 5–10% of the cases (Hoogmartens et al., 2021). With 326 reported, among them the A431E, *PSEN1* has the most pathogenic variants associated with EOAD (Alzforum, 2021).

With a 1292c> a, rs63750083 nomenclature, A431E is in the exon 12 of *PSEN1*, and in the transmembrane region nine, it changes an alanine by glutamic acid and alters its physical–chemical interaction (Landrum et al., 2018; Alzforum, 2021). A431E has the OMIM code 104311.0033 and is associated with EOAD type 3 (McKusick-Nathans Institute of Genetic Medicine JHU, 2019). A431E has complete penetrance (Bateman et al., 2012). According to the guidelines of the American College of Medical Genetics and Genomics and Association of Molecular Pathology (ACMG/AMP), it is classified as pathogenic due to studies indicating, in a moderate range, an effect on Aβ42/Aβ40 levels and a decreased ratio (PS3-M classification criteria), critical functional location (PM1-M) and low frequency or no control (PM2-M), and strong co-segregation (PP1-S) (Alzforum, 2022).

Rogaeva et al. (2001), made the first description of the variant, which was found in five unrelated cases with a family history of AD and onset before 65 years of age. Later, Yescas et al. (2006) identified 12 families and hypothesized a founder effect of A431E. At the same time, Murrell et al. (2006) added an extra 15 independent families with an A431E history.

A431E is one of the three variants in *PSEN1* with the highest number of affected individuals in Latin America (Dumois-Petersen et al., 2020; Llibre-Guerra et al., 2021). The estimated population varies from 381 (Llibre-Guerra et al., 2021) to 301 (Dumois-Petersen et al., 2020), while the number of people at risk ranges from 463 (Llibre-Guerra et al., 2021) to 560 (Dumois-Petersen et al., 2020). Therefore, an increase in A431E carriers is expected, as diagnostic studies and evaluations are still being held mostly by our group.

The distinctive phenotypic feature of A431E is the high frequency of SP (Santos-Mandujano et al., 2020; Llibre-Guerra et al., 2021). A431E is associated with generalized white matter abnormalities which precede SP (Soosman et al., 2016). In addition, Yescas et al. (2006) identified an exclusive motor presentation along with pyramidal signs, myoclonus, and seizures in cases where the onset of EOAD was before the average age of onset. Llibre-Guerra et al. (2021) reported such findings as uncommon in other *PSEN1* variants.

Cases such as the A431E offer the possibility of understanding the disease’s genetic basis and pathology from the early stages (Fuller et al., 2019), which has triggered interest in genotypic and phenotypic characterization of its carriers. As studies involving individuals with a history of A431E continue to expand, it is necessary to have a reference framework regarding the findings of the phenotypic characteristics of this variant.

Previous literature reviews have focused on genetic aspects of EOAD caused by *PSEN1, PSEN2*, and *APP* variants (Tanzi, 2012; Ringman and Coppola, 2013; Ringman et al., 2014; Hoogmartens et al., 2021), and on the study of biomarkers in both cerebrospinal fluid (Ghidoni et al., 2011a,b; Rostgaard et al., 2015; Schindler et al., 2019) and plasma (Blennow et al., 2012). In addition, reviews focused on the clinical heterogeneity of *PSEN1* variants were also published (Larner and Doran, 2006, 2008; Larner, 2013). However, there are no articles specifically focused on reviewing the findings of A431E, which represents a gap in the literature.

In this article, we sought to review studies that include carriers of the A431E variant to describe and characterize findings (clinical, neuroanatomical, neuropathological, neuropsychological, and possible biomarkers) associated with EOAD through a narrative review synthesis with a qualitative approach. We choose an exploratory review to focus on providing scanning of existing knowledge evidence in response to a specific objective (Arksey and O’Malley, 2005).

This scoping review aims to identify and synthesize the characteristics of the A431E variant of *PSEN1* presented in the findings of EOAD-related studies.

## Methods

### Study Design

An exploratory review was chosen to achieve the aim of this study. The search was conducted in accordance with the Preferred Reporting Items for Systematic Reviews and Meta-Analyses (PRISMA) (Page et al., 2021) statement and the guidelines proposed by Arksey and O’Malley (2005). The authors suggest that this type of study aims to quickly provide a general frame of reference for key aspects such as concepts, sources, and types of evidence. It was chosen to “evaluate the extent, range, and nature of the research activity” regarding the A431E variant associated with EOAD, which corresponds to the first of the aims of exploratory studies proposed by the authors (Arksey and O’Malley, 2005, p. 21).

### Search Strategy

In order to identify the articles to be included, the search was conducted in medicine, biomedical, and multiple search field databases: MEDLINE, PubMed, Scopus, WOS (Web of Science), Ovid, BMC (BioMed Central) databases, and the search engine Google Scholar. Research articles were considered if they included the following keywords: A431E AND *PSEN1* OR *PS1* AND ALZHEIMER; although the following search codes were also used A431E AND *PSEN1* OR *PS1* AND ALZHEIMER NOT SPORADIC NOT LATE ONSET. Articles from all databases published from 2001 to September 2021 were considered. Subsequently, duplicates were removed.

### Study Selection/Screening

An initial screening of the research papers’ abstracts was independently conducted by two reviewers (MO-B and YO-R) considering the aim and the inclusion and exclusion criteria of the study.

Research papers included were those fulfilling the following inclusion criteria: (a) articles that report both the presence of the variant and disease; (b) scientific papers with an original contribution; (c) peer-reviewed publications; and (d) papers available in Spanish or English.

Articles were excluded when they met at least one of the following criteria: (a) a review paper; (b) abstracts of posters, conferences, or academic paper works where no access to the full text was available; (c) independent reviews or no peer-reviewed paper; and (d) experimental studies in cellular models. Full texts were also reviewed by the researchers to determine their eligibility by two reviewers (MO-B and YO-R). During this process, disagreements about the inclusion or exclusion of articles were discussed with third and fourth researchers (VJSG and AACA).

As a final criterion, we considered excluding experimental model studies. Articles were identified in which carriers and individuals with a history of the A431E were included along with participants with a history of other variants and even sporadic AD (SAD). Although in many of these papers the authors did not report the results separately or compared between groups, we highlight that in many of these studies the participant samples consisted mainly of people with a history of EOAD or A431E carriers. Therefore, we considered it appropriate to include the studies and, to facilitate the reading of results, separate them into those that included only people with a history of EOAD or A431E carriers, those including, in addition to the variant of interest, (a) other variants in *PSEN1*, (b) *PSEN1* and *PSEN2*, and (c) *PSEN* and *APP*.

### Data Extraction and Analysis

General data extracted from the studies include the following: authors, year of publication, country, study group, type of sample, and stage/type of participant. In addition, information about techniques and instruments used, study aim, and key findings were also extracted.

Data for each article were extracted without distinguishing between (1) study aim, (2) discipline in which the study is framed, (3) significant findings in results, (4) size sample, (5) country of origin of author or group of authors, and (6) authors discipline.

Data extraction was carried out by two of the authors (MO-B and YO-R). The following types of findings were included in this review: beliefs, caregivers, clinical, genetics, neuroanatomical, neuropathology, neuropsychological, biochemical, electrophysiological, and cerebrospinal fluid (CSF) biomarkers.

Analysis and synthesis of the data were performed using a narrative review approach to capture the diversity of findings related to A431E. We consider this method to be the most appropriate given the heterogeneous characteristics of the results. Gaps in the literature were also identified.

## Results

### Selection of Studies

**FIGURE 1:**
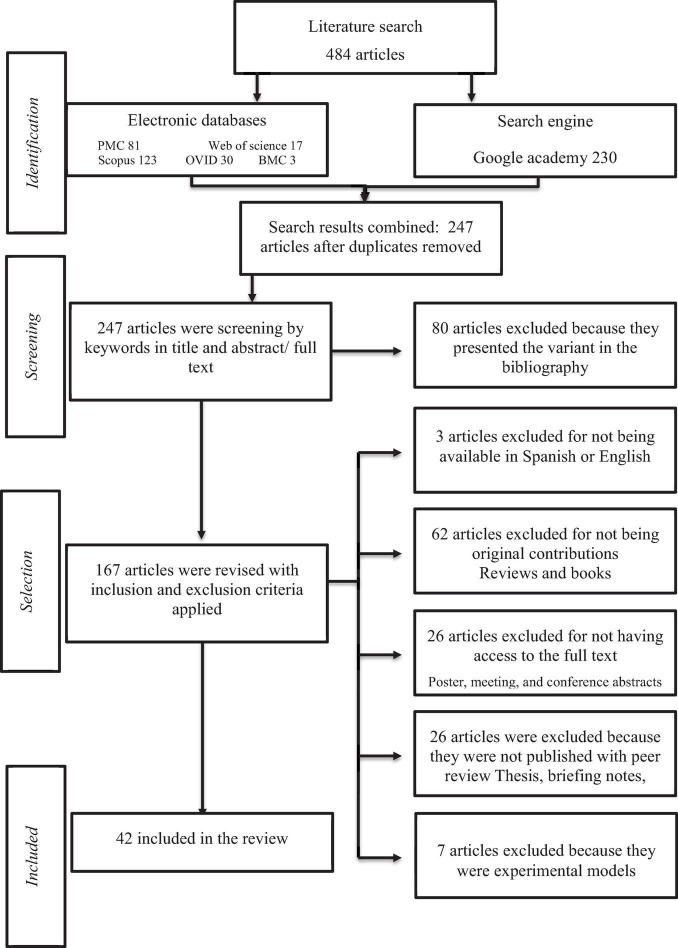
Screening process.

The search has a date range from 2001 to September 2021. We identified a total of 484 articles in five electronic databases and one search engine. After duplicates were removed, a total of 247 articles were left. Following the inclusion criteria, 42 articles were finally considered to be included in this review. The process of identification, screening, eligibility, and inclusion as well as the articles identified in each phase are shown in Figure 1.

### General Characteristics

Since 2001, there has been a gradual increase in the number of publications that consider the A431E *PSEN1* variant (see Table 1).

**TABLE 1:** Summary of general study characteristics.

Study characteristic	Number of studies (*n* = 42)	Percentage
Publication year		
2001–2005	3	7%
2006–2010	12	29%
2011–2015	13	31%
2016–2021	14	33%
Number of participants in studies		
1–10	6	14%
11–20	5	12%
21–30	9	22%
31–40	9	22%
41–50	4	10%
51–60	3	7%
71–80	1	2%
100–200	3	7%
201–500	1	2%
Unknown	1	2%
Not applicable (experimental models)	7	–
Country of origin of the participants		
Latinos (in both Mexico and United States)	40	96%
Germans and Canadians	1	2%
Swedish	1	2%
Stage/participant type		
ADAD family members	2	5%
Preclinical	14	33%
Clinical	5	12%
Preclinical and clinical	14	33%
Preclinical, clinical, and sporadic	1	2%
Postmortem	6	14%
Variants in studies		
A431E	7	17%
*PSEN*	11	26%
FAD	24	57%

*ADAD, autosomal dominant Alzheimer’s disease.*

The number of participants in the studies is also variable, being the studies with the highest percentage of those evaluating c. 21–30 and 31–40 participants. Most of the studies are related to the Latino population, although studies conducted in Germany, Canada, and Sweden have also been performed.

Of the 42 studies, 14 were conducted in a preclinical phase, another 14 in both a preclinical and clinical phase, five were limited to the clinical phase, six of them were postmortem studies, two were in family members at risk for AD, and one included preclinical and clinical participants and individuals with SAD.

### Variants in Studies

Seven of the studies were only on A431E, 11 studies with *PSEN* variants, and the remaining 24 articles are about EOAD [these studies also use the abbreviations FAD (familial Alzheimer’s disease) and autosomal dominant Alzheimer’s disease (ADAD)].

General details of the studies included in this review are presented in Supplementary Table 1, with a detailed study group sample and country, presented accordingly, based on the year of publication. All the studies were conducted in North America, with an exception of two articles in a collaboration with the United States, Canada, and Germany and another in a Swedish publication.

### Category Combinations

The identified 42 studies are all cross-sectional and were classified into several categories according to what was analyzed. Among these, the main ones were beliefs, caregivers, clinical, genetics, studies using imaging techniques, neuropathology, and CSF biomarkers. Such categories are combined between them and with others including neuropsychological, biochemical, and electrophysiological. This classification is presented in Table 2.

**TABLE 2:** Category combinations.

Category combinations		Number of studies	Percentage
		(*n* = 42)	
Beliefs (psychological)		1	2%
Caregivers		1	2%
Clinical	Clinical	1	2%
	Genetics	4	10%
	Genetics, neuropsychological	1	2%
	Genetics, imaging technique	1	2%
Genetics	Genetics	3	7%
	Biomarkers	6	14%
	Linguistic	1	2%
	Imaging technique	5	12%
	Neuropathology	2	5%
	Neuropathology, biochemical	1	2%
	Neuropsychological	3	7%
	Neuropsychological, imaging technique	1	2%
	Electrophysiological	1	2%
Imaging technique	Electrophysiological	1	2%
	Imaging technique	2	5%
	Neuropsychological	2	5%
Neuropathology	Neuropathology	3	7%
	Biochemical	1	2%
CSF Biomarkers		1	2%
Total		42	100%

### Participant Type

Of the 42 studies, two were conducted on members of families with a history of EOAD (Withers et al., 2019, 2021). In the preclinical stage, two of the studies were conducted on *PSEN* variant carriers (Ringman et al., 2004, 2007a) and 12 on subjects with a history or carriers of EOAD-associated variants Ringman et al., 2008a,b, 2011, 2012b,d,e; Golob et al., 2009; Medina et al., 2011, 2021; Braskie et al., 2013; Petok et al., 2018; Joe et al., 2019). In the clinical phase, three of the studies were conducted in A431E carriers (Parker et al., 2019; Alakkas et al., 2020; Dumois-Petersen et al., 2020), and two of the studies were conducted in *PSEN* variant carriers (Joshi et al., 2012; Soosman et al., 2016). Three studies were conducted on A431E carriers in both preclinical and clinical stages (Murrell et al., 2006; Yescas et al., 2006; Santos-Mandujano et al., 2020), “three in *PSEN* variant carriers (Rogaeva et al., 2001; Ringman et al., 2005; Leverenz et al., 2006), and eight in subjects with a history or carriers of EOAD-associated variants (Ringman et al., 2007b,^2010^, 2012a,c; Apostolova et al., 2011; Braskie et al., 2012; Lee et al., 2013; Singer et al., 2021). One study included preclinical and clinical phase participants and individuals with SAD (Portelius et al., 2010). This is classified as part of the studies conducted in A431E carriers as the results were reported per participant. Of the studies conducted on brain tissue, four were performed in *PSEN* variant carriers (Maarouf et al., 2008; Roher et al., 2013; Beck et al., 2016; Gefen et al., 2020) and two in EOAD cases (Albrecht et al., 2009; Ringman et al., 2016).

### Variants Presented in the Studies

#### A431E *PSEN1* Variant

Seven studies are focused on A431E (see Table 3). In 2006, a study hypothesized the founding effect of this variant in the Altos de Jalisco area (Yescas et al., 2006), a letter to the editor, further added 15 independent families to this finding (Murrell et al., 2006). Phenotypic variability reported in the studies, such as SP (Murrell et al., 2006; Yescas et al., 2006; Parker et al., 2019; Dumois-Petersen et al., 2020), motor impairment, visuospatial deficits, olfactory dysfunctions, such as hyposmia and anosmia, as well as respiratory difficulties and visual impairment (Santos-Mandujano et al., 2020), language disorders (Dumois-Petersen et al., 2020), neuropsychiatric symptoms (Alakkas et al., 2020; Dumois-Petersen et al., 2020), atrophy disproportionate to age (Parker et al., 2019; Alakkas et al., 2020; Santos-Mandujano et al., 2020), chronic microhemorrhages within bilateral occipital, temporal, and right frontal lobes and pseudobulbar affect (Parker et al., 2019), and periventricular white matter hyperintensities (Santos-Mandujano et al., 2020), were reported. Two of these studies are case reports (Parker et al., 2019; Alakkas et al., 2020), one of them describing a case of a homozygous person (Parker et al., 2019). In two studies, the average age of dementia onset is mentioned as 42.5 ± 3.9 years (Dumois-Petersen et al., 2020) and 40 years (Yescas et al., 2006). One article identifies low levels of Aβ1–37, Aβ1–38, and Aβ1–39 in cerebrospinal fluid in carriers of this variant, compared to people with SAD (Portelius et al., 2010).

**TABLE 3:** A431E of *PSEN1* studies.

References	Sample	Category	Techniques and instruments used	Study aim	Key findings
Yescas et al., 2006	Carriers with early-onset AD (*n* = 12) and participants with a history of variant (*n* = 85) and healthy controls (*n* = 100).	Genetics	Analysis of microsatellite haplotypes, PCR and MMSE.	Describe a single missense mutation (Ala431Glu) in the *PSEN1* gene found in nine of the 12 apparently unrelated Mexican families with early onset AD.	• The Ala431Glu mutation in exon 12 of *PSEN1* was found in nine (75%) of these families, with autosomal dominant inheritance. • A founder effect was hypothesized. • Microsatellite haplotype analysis suggested a common ancestor in these nine kindreds.
Murrell et al., 2006	15 Families with a history of the A431E variant of *PSEN1.*	Genetics	PCR RFLP.	To expand the observation made by Yescas et al. (2006) by describing an additional 15 independent families with the Ala431Glu substitution in the *PSEN1* gene.	• Additional 15 independent families with the Ala431Glu substitution in the *PSEN1* gene. This mutation is not an uncommon cause of early-onset autosomal dominant AD in persons of Mexican origin.
Portelius et al., 2010	Subjects with sporadic AD (*n* = 18), carriers of the A431E variant in *PSEN1* (*n* = 7), people with depression (*n* = 6) and healthy controls (*n* = 17).	CSF Biomarkers	MMSE, CDR, lumbar puncture, immunoprecipitation analysis and mass spectrometry.	Test the hypothesis that AD is characterized by a specific CSF Ab isoform pattern that is distinct when comparing SAD and FAD due to different mechanisms underlying brain amyloid pathology in the two disease groups.	• Low CSF levels of Aβ1–42 and high levels of Aβ1–16 distinguished SAD patients and FAD mutation carriers from healthy controls and depressed patients. • SAD and FAD were characterized by similar changes in Aβ1–42 and Aβ1–16, but FAD mutation carriers exhibited very low levels of Aβ1–37, Aβ1–38, and Aβ1–39.
Parker et al., 2019	*A431E* (*n* = *1*) *mutation carrier*.	Genetics, Clinical	Family history, MMSE, PCR, and MRI.	Report a 35-year-old male with childhood learning disability and early onset dementia who is homozygous for the A431E variant in the *PSEN1* gene.	• Homozygosity for the A431E variant in *PSEN1.* • Clinical evaluation demonstrated SP and pseudobulbar affect. • Brain MRI revealed cerebral atrophy disproportionate to age. • Chronic microhemorrhages within bilateral occipital, temporal, and right frontal lobes were seen.
Santos-Mandujano et al., 2020	Carriers of the A431E variant in *PSEN1* (*n* = 4), of which one was symptomatic and the rest asymptomatic.	Genetics, clinical, Imaging technique	PCR, MMSE, MOCA, CDR, UPSIT, and MRI.	Characterized three A431E mutation carriers, one symptomatic and two asymptomatic, from a Mexican family with a history of SP in all its affected members.	• Symptomatic subject showed an atypical non-amnestic mild cognitive impairment with visuospatial deficits, olfactory dysfunction, and significant parieto-occipital brain atrophy. • Several periventricular white matter hyperintensities whose progression pattern and localization correlated with their motor impairment.
Dumois-Petersen et al., 2020	Of the total number of participants (*n* = 54), *n* = 46 were carriers of the A431E variant.	Genetics, Clinical	PCR, Neuropsychiatric Inventory Questionnaire.	Present the initial evaluation of 46 individuals with AD-EOAD, all of whom from the Mexican state of Jalisco and carrying the A431E mutation in the *PSEN1* gene.	• The mean onset age was 42.5 ± 3.9 years. • Substantial clinical heterogeneity and high frequencies of SP, language disorders, and neuropsychiatric symptoms.
Alakkas et al., 2020	*n* = *1 Woman with variant in PSEN1*.	Clinical	Electroencephalogram, Bush–Francis scale of Catatonia, clinical history, MRI, laboratory testing, genetic testing.	Report a case of a 35-year-old woman with significant deterioration in psychomotor functioning, depression, and catatonic features.	• This case is a cautionary reminder for clinicians that end stages of dementia can present similar to catatonia with mutism, lack of spontaneous movement, and refusal to eat. • The clues to the diagnosis were profound cortical atrophy and lack of improvement with optimal medical management.

*Ab, amyloid β protein; AD, Alzheimer’s disease; AD-EOAD, autosomal dominant early−onset Alzheimer’s disease; CDR, Clinical Dementia Rating Scale; CSF, cerebrospinal fluid; FAD, familial Alzheimer’s disease; MMSE, Mini-Mental State Examination; MOCA, Montreal Cognitive Assessment; MRI, magnetic resonance imaging; PCR, polymerase chain reaction; RFLP, restriction fragment length polymorphism analysis; SAD, sporadic Alzheimer’s disease; SP, spastic paraparesis; and UPSIT, University of Pennsylvania Smell Identification Test.*

We found A431E in studies mixed with other genetic variants; in Table 4, we classified the articles analyzing *PSEN* variants. Eight studies focused on *PSEN1* variants (Rogaeva et al., 2001; Ringman et al., 2004, 2005, 2007a; Joshi et al., 2012; Beck et al., 2016; Soosman et al., 2016; Gefen et al., 2020) and, in three more, variants in *PSEN1* and *PSEN2* are analyzed (Leverenz et al., 2006; Maarouf et al., 2008; Roher et al., 2013).

**TABLE 4:** *PSEN* studies.

References	Sample	Category	Techniques and instruments used	Study aim	Key findings
**Studies whit variants in *PSEN1***					
Rogaeva et al., 2001	Total number of participants (*n* = 414), *n* = 372 had AD and *n* = 42 asymptomatic with a history. *n* = 48 of the participants had *PSEN1* variants, the most frequent being Glu206Ala (*n* = 25), Ala431Glu (*n* = 25) and Ile143Thr (*n* = 3), in addition, 21 new variants were identified.	Genetics	PCR	Report the experience of mutation screening in a series of consecutive patients with AD referred for diagnostic testing.	• Forty-eight independent patients screened had a *PS1* mutation including 21 novel mutations. • The majority of the mutations were missense substitutions.
Ringman et al., 2004	Of the total number of participants (*n* = 33), there were subjects from families with a history of the A431E variant (*n* = 22) between these carriers (*n* = 12) and non-carriers (*n* = 10). In addition to subjects with a history of L235V (*n* = 11) between these carriers (*n* = 5) and non-carriers (*n* = 6).	Genetics, clinical, neuropsychological	MMSE, Spanish Cognitive test, CDR, BDI, PCR RFLP.	To study depressive symptoms in preclinical *PS1* related Alzheimer’s disease.	• Depressive symptoms can occur early in the course of *PS1* related Alzheimer’s disease, at least in women. • Not demented mutation carriers tended to score lower than non-carriers on several neuropsychological tests.
Ringman et al., 2005	Participants without dementia with a history of variants in *PSEN1* (*n* = 51). carriers of A431E (*n* = 25), and L235V (*n* = 5) non-carriers (*n* = 21), of which 15 had a history of A431E and 6 of L235V.	Genetics, neuropsychological	MMSE, BDI, TMT (forms A and B), WMS-R, Rey Osterrieth Figure, 10-Word Learning List (immediate and delayed retrieval), Boston naming test, verbal fluency (semantics [fruits and animals] and phonological [F, A]), WAIS cube design and PCR.	To investigate these observations by the study of persons at risk for autosomal dominant forms of AD.	• Early problems with memory, visuospatial function, and particularly with executive function in *PS1* mutation carriers. • Depression, gender, and presence of an *APOE*ε4 allele did not demonstrate large influences on neuropsychological performance.
Ringman et al., 2007a	The participants were Mexicans (*n* = 50), who had a history of the A431E (*n* = 39) and L235V (*n* = 11) mutations, of which *n* = 29 were carriers and *n* = 21 were non-carriers.	Genetics, neuropsychological	Clinical interview, PCR RFLP, computerized version of MMSE.	Explore the sub-items on the MMSE that best differentiate *PSEN1* MCs and NCs and explore the relationship of age and education to these scores.	• Subjects in the earliest stage of *PSEN1*-related AD showed deficits on orientation to date and in divided attention when spelling backward. • The relative lack of deficits on delayed recall of three words probably represents the insensitivity of this measure in early AD. • This study supports the utility of ADAD as a model of the more common sporadic form of the disorder.
Joshi et al., 2012	Subjects with familial AD associated with *PSEN1* (*n* = 32), of which *n* = 22 had the A431E variant, in addition the sample consisted of carriers of the following variants G206A (*n* = 2), L235V (*n* = 3), M146L (*n* = 1), S212Y (*n* = 1), R269H (*n* = 1), I238M (*n* = 1), and T245P (*n* = 1). Subjects with unfamiliar early-onset AD (*n* = 81). Results are not specified by mutation.	Clinical	MMSE and medical history review.	To identify clinical features that distinguish FAD from non-familial EAD.	• FAD patients with *PSEN1* mutations were more likely to have significant headaches, myoclonus, gait abnormality, and pseudobulbar affect. • Differences in pathophysiology between FAD and NF-EAD and findings in some contexts should lead to genetic counseling and appropriate recommendations for genetic testing for FAD.
Soosman et al., 2016	Carriers of variant A431E with paraparesis (*n* = 3) and carriers of variants A431E (*n* = 1), G206A (*n* = 2), I238M (*n* = 1), M146L (*n* = 1), R269H (*n* = 1), S212Y (*n* = 1) without paraparesis. All these variants are in *PSEN1*.	Electrophysiological, Imaging technique	MMSE, CDR, diffuser tensor, MRI, volumetric analysis, microbleed counting, amyloid PET using PiB and somatosensory and motor evoked potential studies and electrophysiological studies.	Compared diffusion and volumetric magnetic resonance measures between 3 persons with SP associated with the A431E mutation and 7 symptomatic persons with *PSEN1* mutations without SP matched for symptom duration.	• Decreases in FA and increases in mean diffusivity in widespread white matter areas including the corpus callosum, occipital, parietal, and frontal lobes in *PSEN1* mutation carriers with SP. • Volumetric measures were not different and amyloid imaging showed low signal in sensorimotor cortex and other areas in a single subject with SP. • Electrophysiological studies demonstrated both slowed motor and sensory conduction in the lower extremities.
Beck et al., 2016	Postmortem samples of frontal tissue (Brodmann’s area 10) of cognitively intact controls (*n* = 9), sporadic-type AD (*n* = 8) and of the variants T115C, I143T, G209V, A260V, A431E (*n* = 8) of *PSEN1*.	Genetics, neuropathology	PCR and Western Blot.	To test whether mtUPR activation occurs in AD, we performed real-time quantitative PCR on postmortem frontal cortex samples from subjects classified as sporadic AD, familial AD linked to presenilin-1 mutations, or cognitively intact controls.	• Levels of all six mtUPR genes were significantly up-regulated by ∼70–90% in familial AD.
Gefen et al., 2020	Brain tissue of subjects with PPA (*n* = 16). Behavioral variant of frontotemporal dementia (*n* = 16). Alzheimer’s type dementia (*n* = 16), of the latter two had variant H163R (*n* = 1) and A431E (*n* = 1) associated with *PSEN1*.	Neuropathology	Semiquantitative counting method to measure the degree of macroscopic atrophy, neuronal loss, and gliosis, superficial microvacuolation and pathological inclusions.	Determine whether leftward asymmetry is unique to PPA compared with the typical dementia of the Alzheimer’s type and bvFTD.	• PPA has an exclusive pathologic signature, distinct from DAT and bv FTD, with PPA favoring the language-dominant hemisphere, typically left. This unique signature was consistent across all examinations of gross pathology, neuronal loss and gliosis, and microvacuolation, particularly in the temporal region.
**Studies whit variants in *PSEN1* and *PSEN2***					
Leverenz et al., 2006	Cases with *PSEN1* variants (*n* = 25), including: A260V (*n* = 7), G209V (*n* = 7), E120D (*n* = 3), A431E (*n* = 2), M233L (*n* = 2), H163R (*n* = 1), I143T (*n* = 1), L418F (*n* = 1) y M146L (*n* = 1). *PSEN2* variant N141I cases (*n* = 14).	Genetics, neuropathology	Lewy body neuropathology was examined using synuclein immunohistochemistry and sampling of multiple brainstem and cortical regions, and PCR.	To examine LBP in the brainstem, limbic cortex, and neocortex of a large number of familial AD cases with mutations in 2 *PSEN* genes.	• The amygdala was the most vulnerable site for LBP in *PSEN1* mutation cases. Genetic influences on the presence of LBP in familial AD as demonstrated by the differences between *PSEN1* and *PSEN2* mutation cases.
Maarouf et al., 2008	Brain tissue from carriers of *PSEN1* (*n* = 9) including: A79V (*n* = 1), A260V (*n* = 1), F105L (*n* = 1), Y115C (*n* = 1), A431E (*n* = 1), V261F (*n* = 1), V261I (*n* = 1), M146L (*n* = 1), P264L (*n* = 1); N141I of *PSEN2* (*n* = 1) carrier; subjects with sporadic AD (*n* = 4), and healthy controls (*n* = 2).	Genetics, neuropathology, biochemical	PCR, Western Blot, densitometry scanning and ELISA.	Compare neuropathological and biochemical findings among nine independent *PSEN1* and one *PSEN2* FAD cases, four SAD cases and two non-demented controls, determined Aβ40 and Aβ42 peptide levels, and the processing pattern and relative quantities of AβPP N-terminal and C-terminal peptides and soluble tau and investigated the differences among the *PSEN* mutations, as well as between the *PSEN* group and SAD or ND controls, with respect to Notch-1, N-cadherin and Erb-B4, molecules that are cleaved by the γ-secretase complex.	• Missense mutations in *PSEN* genes can alter a range of key γ-secretase activities to produce an array of subtly different biochemical, neuropathological, and clinical manifestations.
Roher et al., 2013	Brain tissue (white matter) of *PSEN1* variant carriers: A79V (*n* = 1), F105L (*n* = 1), Y115C (*n* = 1), M146L (*n* = 1), A260V (*n* = 1), V261F (*n* = 1), V261I (*n* = 1), P264L (*n* = 1), and A431E (*n* = 1); and substitution carriers in *PSEN2*: N141I (*n* = 1).	Neuropathology, biochemical	Western Blot and ELISA.	Examine the WM biochemistry by ELISA and Western blot analyses of key proteins in 10 FAD cases harboring mutations in the presenilin genes *PSEN1* and *PSEN2* as well as in 4 non-demented control individuals and 4 subjects with SAD.	• The *PSEN*-FAD mutations we examined did not produce uniform increases in the relative proportions of Aβ42 and exhibited substantial variability in total Aβ levels. Additional complexities in *PSEN*-FAD individuals. • Some direct substrates of γ-secretase, such as Notch, N-cadherin, Erb-B4 and *APP*, deviated substantially from the NDC group baseline for some, but not all, mutation types.

*AD, Alzheimer’s disease; ADAD, autosomal dominant Alzheimer’s disease; BDI, Beck Depression Inventory; bvFTD, behavioral variant frontotemporal dementia; CDR, Clinical Dementia Rating Scale; DAT, dementia of the Alzheimer’s type; EAD, Early-onset AD; FA, fractional anisotropy; FAD, familiar Alzheimer’s disease; LBP, Lewy body pathology; MCs, mutation carriers; MMSE, Mini-Mental State Examination; mtUPR, mitochondrial unfolded protein response; NCs, non-carriers; ND, non-demented control; NDC, non-demented control; NF-EAD, non-familial EAD; PCR, polymerase chain reaction; PET, positron emission tomography; PiB, Pittsburgh Compound B; PPA, primary progressive aphasia; RFLP, restriction fragment length polymorphism analysis; SAD, sporadic Alzheimer’s disease; SP, spastic paraparesis; TMT, Trail Making Test; WAIS, The Wechsler Adult Intelligence Scale; WM, white matter; and WMS-R, Wechsler Memory Scale–Revised.*

#### PSEN1

One article in the Genetics category described novel missense variant substitutions (Rogaeva et al., 2001), among them, is the Ala431Glu. In four studies, the authors identified clinical and neuropsychological characteristics in the participants with variants in *PSEN1*. In the early stages, memory, visuospatial, and executive function deficits were found (Ringman et al., 2005). In a study using the Mini-Mental State Examination (MMSE), a decreased temporal orientation performance and divided attention were observed, whereas the three-word list subtest has no sensitivity in identifying changes in memory in the preclinical stage (Ringman et al., 2007a). In the case of women, depressive symptoms were identified (Ringman et al., 2004), and carriers of *PSEN1* mutations in the clinical stage more significantly presented headaches, myoclonus, gait abnormality, and pseudobulbar affect (Joshi et al., 2012). In these articles, the sample is composed primarily of people with the A431E variant. One article reported findings related to electrophysiological and imaging techniques in carriers with and without SP, where SP carriers showed decreased fractional anisotropy, increased mean diffusivity in widespread white matter areas, and slow motor and sensory conduction in the inferior extremities (Soosman et al., 2016).

Two articles study brain tissue of carriers of *PSEN1* variants, and one presents a significant upregulation of six genes related to mitochondrial unfolded protein response (Beck et al., 2016) with primarily differences between primary progressive aphasia (PPA), AD, and a behavioral variant of frontotemporal dementia (Gefen et al., 2020).

#### *PSEN1* and *PSEN2*

Three studies presented neuropathological features; differences among these genes were found in one article: in *PSEN1* mutation cases, the amygdala was more vulnerable to Lewy body pathology than in *PSEN2* (Leverenz et al., 2006). The other two analyze the biochemical and neuropathological implications (Maarouf et al., 2008; Roher et al., 2013), variation in levels of beta-amyloid, and differences in some substrates of gamma secretase (Roher et al., 2013) along with its dysfunction (Maarouf et al., 2008).

After presenting the studies exclusively to *PSEN*, we categorized those who reported in combination with *APP*. In Table 5, we present these articles in three categories: first, articles with *PSEN1* and *APP* (Ringman et al., 2007b, 2008a,b, 2010, 2011, 2012a,b,c,d,e; Golob et al., 2009; Apostolova et al., 2011; Braskie et al., 2012, 2013; Lee et al., 2013; Joe et al., 2019; Medina et al., 2021; Singer et al., 2021); second, articles with *PSEN1, PSEN2*, and *APP* variants (Albrecht et al., 2009; Medina et al., 2011; Ringman et al., 2016; Petok et al., 2018), and finally, those articles who reported families with a history of EOAD (Withers et al., 2019) and another with A431E *PSEN1* and an unknown mutation (Withers et al., 2021).

**TABLE 5:** *PSEN* and *APP* studies.

References	Sample	Category	Techniques and instruments used	Study aim	Key findings
**Studies whit variants in *PSEN1* and *APP***					
Ringman et al., 2007b	Total number of participants (*n* = 23), *n* = 12 were carriers and *n* = 8 were non-carriers, among these *n* = 19 had a history of variants in *PSEN1* (including A431E) and *n* = 4 in *APP*. No quantities are specified.	Genetics, Imaging technique	CDR, MMSE, Diffuser Tensor, MRI, and PCR.	Compare global and localized fractional anisotropy measures in WM between FAD mutation carriers and non-carriers in the preclinical and presymptomatic stages of the disease.	• FA is decreased in the WM in preclinical and even presymptomatic FAD mutation carriers, particularly in the late-myelinating tracts connecting limbic structures. • Decreased FA in of the columns of the fornix is particularly robust in early FAD.
Ringman et al., 2008a	The participants (*n* = 27) were divided into carriers (*n* = 15) and non-carriers (*n* = 12), who came from 11 families, of which 9 had a history of variants in *PSEN1* (7 from A431E, one from L235V and another of G206A) and 2 of variants in *APP* (the results do not distinguish between carriers with different variants).	Genetics, clinical	Structured interview based on the ICHD-2; CDR; PCR RFLP.	Compare the prevalence of headaches between non-demented FAD MCs and NCs controls.	• The tendency for a higher prevalence of headaches in MCs held for different *PSEN1* and *APP* mutations but was not significant unless all families were combined. • Headache was more common in non-demented FAD MCs than NCs. Possible mechanisms for this include cerebral inflammation, aberrant processing of Notch3, or disrupted intracellular calcium regulation.
Ringman et al., 2008b	The participants (*n* = 21) were carriers of variants in *PSEN1* (*n* = 17) and in *APP* (*n* = 4). They came from families with variants in A431E (*n* = 4), L235V (*n* = 1), G206A (*n* = 1) of *PSEN1*, and families with V717I variants (*n* = 2) of *APP*.	Genetics, biomarkers	CDR, MMSE, ELISA, and PCR.	Measured levels of plasma (Aβ40, Aβ42, F2-isoprostanes) and CSF (F2- isoprostanes, t-tau, p-tau181, Aβ40, and Aβ42) biomarkers with putative relationships to AD status and progression in persons at risk for FAD to help clarify these relationships.	• Aβ42 is elevated in plasma in FAD MCs and suggests that this level may decrease with disease progression prior to the development of overt dementia. • The ratio of Aβ42 to Aβ40 was reduced in the CSF of non-demented MCs and that elevations of t-tau and p-tau181 are sensitive indicators of presymptomatic disease. • Elevated F2-isoprostane levels in the CSF of preclinical FAD MCs suggests that oxidative stress occurs downstream to metabolism of amyloid precursor protein.
Golob et al., 2009	Participants with a history of variants associated with *PSEN1* (*n* = 19), including a history of A431E (*n* = 14) and L235V (*n* = 5); and associated with *APP* V717I substitution (*n* = 5). Variant carriers in *PSEN1* and *APP* are not separated in the results.	Genetics, electrophysiological	Genetic testing, CDR, MMSE, CASI, PCR RFLP; target detection oddball task of listening to a sequence of tones at 2.5 s intervals; ERP.	To define changes in cortical function in persons inheriting FAD mutations before the onset of cognitive decline.	• FAD mutation carriers had significantly longer latencies of the N100, P200, N200, and P300 components, and smaller slow wave amplitudes. • Auditory sensory and cognitive cortical potentials in persons with FAD mutations are abnormal approximately 10 years before dementia will be manifest.
Ringman et al., 2010	Participants with dementia (*n* = 4), mild symptoms (*n* = 7), and asymptomatic (*n* = 28) from 13 families carrying variants in *APP* (*n* = 2) or *PSEN1* (*n* = 11). Among families with a history of *PSEN1* variants, one had L235V, one G206A, one S212Y, and eight had the A431E substitution. Number of carriers is not specified.	Imaging technique	CDR and MRI.	To assess the ability of radiologists to detect HA in persons destined to develop AD.	Radiologists’ ability to detect HA in persons in whom the diagnosis of incipient AD is certain is suboptimal and quantitative MRI techniques or other biological markers of the disease are needed.
Ringman et al., 2011	The participants (*n* = 23) came from families with a history of *PSEN1* variants, including: 1 with a history of L235V and 5 families with A431E. While the rest of the subjects belong to two families with a history of the V717I variant of *APP*. Of the total number of participants, *n* = 14 were carriers and *n* = 9 were non-carriers. The number of carrier and non-carrier participants per variant is not specified.	Genetics, neuropsychological, imaging technique	PCR RFLP, MRI, semantic verbal fluency test (animals), naming of objects, rey figure, WAIS cube design, word list retrieval and Stroop.	To study the effects of FAD mutation status and *APOE* genotype on fMRI activation during a novelty encoding task in a larger number of presymptomatic subjects at-risk for FAD mutations to differentiate the effects of these genes.	• FAD MCs (*n* = 14) showed decreased BOLD activation in the anterior cingulate gyrus relative to 9 NCs. • No increased activation was seen in MCs relative to NCs. • Increased fMRI activation associated with *APOE* genotype but not with FAD mutations.
Apostolova et al., 2011	Control subjects (*n* = 11). Carriers without dementia (*n* = 22) of variants A431E (*n* = 14), L235V and G206A (*n* = 3) associated with *PSEN1* and the V717I *APP* variant (*n* = 5). Carriers with dementia (*n* = 3), A431E (*n* = 1), L235V and G206A (*n* = 2).	Genetics, Imaging technique	MMSE, CDR, PCR RFLP, and MRI.	Define cortical and hippocampal atrophy in an independent cohort of persons at risk for FAD using different structural MRI analytical techniques.	• FAD is associated with thinning of the posterior association and frontal cortices and HA. • arger sample sizes may be necessary to reliably identify cortical atrophy in presymptomatic carriers.
Ringman et al., 2012a	Non-carriers (*n* = 5). Presymptomatic subjects (*n* = 10), including carriers of the A431E (*n* = 7) and L235V (*n* = 1) variants of *PSEN1* and the V717I variant (*n* = 2) of *APP*. Symptomatic carriers (*n* = 4), including carriers of the A431E (*n* = 2), L235V (*n* = 1) and S212Y (*n* = 1) variants of *PSEN1*.	Genetic, Biomarkers	CDR, PCR RFLP, and Lumbar puncture, mass spectrometry analysis, with ion trap analyzer.	To identify CSF protein changes in persons who will develop FAD due to *PSEN1* and *APP* mutations, using unbiased proteomics.	• Overlap in CSF protein changes between individuals with presymptomatic and symptomatic FAD. • Inflammation and synaptic loss early in FAD and suggest new presymptomatic biomarkers of potential usefulness in drug development.
Ringman et al., 2012b	Carriers (*n* = 21) and non-carriers (*n* = 12) from families with history of *PSEN1* variants (12 families, including 9 with A431E variant, 1 of L235V, 1 of G206A y 1 of S212Y) and *APP* (2 families with history of V717I variant).	Genetics, biomarkers	CDR, and PCR RFLP.	To study the effect of FAD mutations and *APOE* genotype on plasma signaling protein levels.	• Different patterns of inflammatory markers in young and middle-aged persons among *APOE* genotype groups. • The *APOE* ε4 carriers had the lowest levels of apolipoprotein E. • Young ε4 carriers have increased inflammatory markers that diminish with age.
Ringman et al., 2012c	Participants (*n* = 31) with a history of *PSEN1* mutations (*n* = 23) from 10 families, of which 14 had the mutation, *n* = 8 with A431E mutation, *n* = 1 with G206A, *n* = 1 with S212Y (does not mention the mutation of the rest of participants). Participants with a history of *APP* (*n* = 8) from 2 families with variant V7171 of which *n* = 5 were carriers. Of the total number of participants, *n* = 19 were carriers (*n* = 14) of *PSEN1* variants and *n* = 5 of *APP* variants, while *n* = 12 were non-carriers.	Genetics, biomarkers	CDR, western blot with a polyclonal anti-MetO antibody, isoprostane measurement, multiple protein analysis and PCR RFLP.	To ask if oxidation of methionine residues to methionine sulfoxide was increased in plasma proteins of persons carrying FADmutations.	Elevated MetO levels in persons carrying FAD mutations that correlate with other indices of oxidative stress.
Ringman et al., 2012d	Participants (*n* = 7) were divided into asymptomatic carriers (*n* = 5) and non-carriers (*n* = 2). Participants had a history of L235V and A43E mutations in *PSEN1*, and V7171 in *APP* (specific mutations were not disclosed due to confidentiality).	Genetics, biomarkers	CDR, PCR RFLP, CSF with dot-blot using polyclonal antibodies A11 (anti-prefibrillar oligomer), OC and αAPF AB42 levels in CSF by ELISA; protein concentration was determined using the BCA Protein Assay Kit.	To identify oligomers during the presymptomatic stage of the disease in persons destined to develop FAD.	Evidence for an identifiable elevation of CSF oligomers during the presymptomatic phase of FAD.
Ringman et al., 2012e	Carriers (*n* = 13) and non-carriers (*n* = 5). Of the carriers, *n* = 11 had variants associated with *PSEN1* and *n* = 2 associated with *APP*. Among the variants associated with *PSEN1*, the participants had a history of A431E, L235V and S212Y, however, the number of carriers and non-carriers of this variant was not specified.	Genetics, biomarkers	MMSE and CDR. CSF analysis, innogenetics INNO-BIA AlzBio3 multiplex assays were used in standardized xMAP Luminex technology, and PCR RFLP.	Evaluate changes in CSF levels of 42-amino-acid β-amyloid (Aβ42), total tau protein (t-tau) and phosphorylated tau at residue 181 (p- tau181).	• There was a negative correlation between Aβ42 levels and age relative to the family-specific age of dementia diagnosis. • A decline in CSF Aβ42 levels occurring at least 20 years prior to clinical dementia in FAD.
Braskie et al., 2012	Subjects were asymptomatic or had mild cognitive impairment. Carriers (*n* = 18) and non-carriers (*n* = 8) had a history of the A431E and L235V variants of *PSEN1* and V717I of *APP*. Carrier variants are not specified.	Neuropsychological, imaging technique	CDR, MMSE, CASI, verbal fluency, Stroop, word list recall and fMRI.	Compared fMRI activity of non-demented autosomal dominant AD mutation carriers with fMRI activity in their non-carrier relatives as they performed a novelty encoding task in which they viewed novel and repeated images.	• Mutation carriers showed increased fMRI activity in the fusiform and middle temporal gyri. • During novelty encoding, increased fMRI activity in the temporal lobe may relate to incipient AD processes.
Braskie et al., 2013	Carriers (*n* = 9), of these *n* = 8 had the A431E variant of *PSEN1* while *n* = 1 had the V717I of *APP*, and non-carriers (*n* = 8).	Imaging technique	MMSE, CASI and fMRI performing a memory task.	Examine fMRI signal differences between carriers and non-carriers, and how signal related to fMRI task performance within mutation status group, controlling for relative age and education.	Poorer performing carriers showed greater retrieval period signal, including in the frontal and temporal lobes, suggesting underlying pathological processes.
Lee et al., 2013	Of the total number of participants (*n* = 35), *n* = 25 were carriers and *n* = 10 were non-carriers. Of the carriers, *n* = 21 had *PSEN1* A431E mutation and *n* = 4 *APP*-associated mutations. The results are not separated according to the mutation to respect the confidentiality of the participants.	Genetics, neuropsychological, imagine technique	CDR, MMSE, word list learning, MVP, Rey-Osterrieth figure, digit-symbol, Stroop, block design, category fluency, test object naming, WCST and MRI, PCR RFLP.	Examined brain volume differences between presymptomatic and symptomatic FAD mutation carriers and non-carrier relatives using tensor-based morphometry.	Cognitively intact FAD mutation carriers had lower thalamic, caudate and putamen volumes, and there is preliminary evidence for increasing caudate size during the predementia stage. These regions may be affected earliest during prodromal stages of FAD, while cortical atrophy may occur in later stages, when carriers show cognitive deficits.
Joe et al., 2019	Of the total number of carriers (*n* = 16), *n* = 11 had the A431E variant and *n* = 2 L235V of *PSEN1*, while of *APP n* = 3 had the V717I variant. Non-carriers (*n* = 11) were subjects with a history of the A431E (*n* = 7), L235V (*n* = 2) variants of *PSEN1* and V717I (*n* = 2) of *APP*.	Genetics, imaging technique	MRI, MRS, CDR, and PCR.	Attempted to identify changes in levels of metabolites prior to the onset of clinical symptoms in carriers of ADAD mutations.	• MCs had significantly lower levels of NAA and Glx in the left pregenual anterior cingulate cortex, and lower levels of NAA and higher levels of mI and Cho in the precuneus. • Increased levels of mI were seen in these regions in association with increased proximity to expected age of dementia onset.
Medina et al., 2021	The sample of participants (*n* = 71) was divided into carriers (*n* = 40) and non-carriers (*n* = 31). Carriers had the following variants: A431E (*n* = 29) and L235V (*n* = 7) of *PSEN1*, and V717I (*n* = 4) of *APP*. While non-carriers had a history of the following variants A431E (*n* = 20), L235V (*n* = 7) and G206A (*n* = 1) of *PSEN1*, and V717I (*n* = 3) of *APP*.	Genetics, neuropsychological	Cognometer computer program including time reaction tasks, PCR RFLP.	Evaluate attention and working memory using a computerized battery in non-demented persons carrying ADAD mutations.	• MCs respond more slowly as they approach the age of dementia onset on tasks with greater demands on executive function. These effects were not explained by *APOE*ε*4* status independently of ADAD mutation status. • Computerized reaction time tests can provide sensitive measures of the earliest cognitive changes in AD.
Singer et al., 2021	Of the total number of participants (*n* = 39), some had variants in *PSEN1*, including A431E (*n* = 11) and F388S (*n* = 1), while the rest of the carriers had variant V717I (*n* = 1) of *APP* and the rest of the participants (*n* = 21) were controls with a history of the previous variants.	Genetics, imaging technique	OCTA imaging protocol-quantitative capillary flow and morphometric, and PCR.	Characterize retinal capillary blood flow in subjects with ADAD-causing mutations.	Increased perfusion is a pathophysiologic feature of presymptomatic stages of ADAD.
**Studies whit variants in *PSEN1*, *PSEN2*, and *APP***					
Albrecht et al., 2009	Brain tissue of cases with the variants *APP* K670N, M671L (*n* = 5), *APP* E693G (*n* = 1), *PSEN1* M146V (*n* = 2), *PSEN1* A431E (*n* = 2), *PSEN1* F105L (*n* = 2), *PSEN1* V261F (*n* = 2) *PSEN1* Y115C (*n* = 2) and *PSEN2* N141I (*n* = 1).	Neuropathology	Immunoreactivity with anti-active Casp-6 and Tau cleaved by Casp-6 y Semiquantitative Scoring of Immunostaining.	To determine if Casp-6 is activated in familial AD.	• Active Casp-6 immunoreactivity was found in all cases. • Caspase-6 immunoreactivity was observed in neuritic plaques or in some cases cotton-wool plaques, and in neuropil threads and neurofibrillary tangles.
Medina et al., 2011	Subjects without dementia (*n* = 35) with a history of variants associated with familial AD. Of these *n* = 30 had a history of *PSEN1* variants, *n* = 1 of *PSEN2* and *n* = 4 of *APP*.	Genetics, linguistic	CDR, PCR RFLP, CASI and writing biographical essays to determine propositional density (relationship between the number of unique ideas and the number of words in the text).	To explore the relationship between FAD mutation status, *APOE* genotype, and p-density.	• FAD mutation status was not significantly associated with p-density. • *APOE* ε4 carriers having lower p-density than non-carriers.
Ringman et al., 2016	Neuropathologic (postmortem) data of cases with variants in *PSEN1* (*n* = 46), including A79V (*n* = 5), I143T (*n* = 2), M233L (*n* = 2), Y115C (*n* = 2), M146L (*n* = 2), L235V (*n* = 1), Y115H (*n* = 1), Y156insFI (*n* = 1), T245P (*n* = 2), E120D (*n* = 1), H163R (*n* = 4), V261F (*n* = 1), N135D (*n* = 1), S170F (*n* = 1), P267A (*n* = 1), N135S (*n* = 2), G206A (*n* = 6), A431E (*n* = 6), M139I (*n* = 1), G209V (*n* = 1), L435F (*n* = 1), M139V (*n* = 1) y L226R (*n* = 1). *APP* (*n* = 10), including E693G (*n* = 1), V717I (*n* = 3), V717F (*n* = 4) y V717L (*n* = 2). N141I in *PSEN2* (*n* = 4).	Neuropathology	CERAD and semi-quantification of diffuse and neuritic amyloid plaques on a scale of 0 to 3 (none, scarce, moderate, or frequent) in frontomedial, temporal, and inferior parietal regions.	Compare hallmark AD pathologic findings in 60 cases of ADAD and 120 cases of sporadic AD matched for sex, race, ethnicity, and disease duration.	• The finding of Lewy body pathology in a substantial minority of ADAD cases supports the assertion that development of Lewy bodies may be in part driven by abnormal b-amyloid protein precursor processing. • In persons with *PSEN1* mutations beyond codon 200 had higher average Braak scores and severity and prevalence of CAA.
Petok et al., 2018	Of the total number of participants (*n* = 45), *n* = 34 were carriers and *n* = 11 were non-carriers. Of these *n* = 13 had a history of variant V717I of *APP*, *n* = 12 of variant A431E, *n* = 5 of G206A, *n* = 4 L235V, *n* = 3 R269H, *n* = 1 A260V, *n* = 1 E184D, *n* = 1 E280A, *n* = 1 H163R, *n* = 1 S212Y, *n* = 1 C410Y, *n* = 1 G378E of *PSEN1*. While *n* = 1 had a history of *PSEN2* variant N14I.	Genetics, imaging technique	MMSE, CASI, PCR, generalization task on SuperCard and MRI.	Compared preclinical individuals carrying ADAD mutations to non-carrying kin to determine whether generalization (the ability to transfer previous learning to novel but familiar recombinations) is vulnerable early, before overt cognitive decline.	Preclinical ADAD mutation carriers made significantly more errors during generalization. This impairment correlated with left hippocampal volume, particularly in mutation carriers.
**A431E and unknown mutation**					
Withers et al., 2021	The participants (*n* = 27) came from families with a history of variants associated with early-onset AD. Five focus groups were held for discussion, of which four were conducted with people whose relatives are carriers of the A431E *PSEN1* variant, while the variant was not determined in the fifth focus group.	Caregivers	Demographic Survey, CBAD, ADKS and the Zarit Caregiver Burden Scale, focus groups.	To explore the experiences and needs of Latino caregivers of persons with EOAD.	• The stress of caregiving was compounded by other pressures and worries, such as taking care of young children, providing financially for family, caregivers’ own co-morbidities, and contemplating their own risk of inheriting EOAD. • Resources for monolingual Spanish speakers were scarce. • Difficulty in obtaining a diagnosis from physicians who were uninformed about EOAD was also common.
**ADAD families**					
Withers et al., 2019	*n* = 86 relatives of Mexican patients and *n* = 37 Mexican Americans with ADAD (the CBAD scale was applied) and *n* = 18 surveyed in Mexico.	Beliefs (psychological)	CBAD and semi-structured interview.	Examine cultural beliefs about AD and genetic screening among at-risk populations of Mexican heritage.	The interviews demonstrated that very few at-risk respondents understood their own risk for harboring the mutation causing AD in their family. Once informed, most expressed a strong interest in genetic testing, largely motivated by the desire to be better prepared for the development of AD.

*AD, Alzheimer’s disease; ADAD, autosomal dominant Alzheimer’s disease; ADKS, Alzheimer’s Disease Knowledge Scale; CAA, cerebral amyloid angiopathy; CASI, Cognitive Abilities Screening Instrument; CBAD, Scale of Cultural beliefs about Alzheimer’s Disease; CDR, Clinical Dementia Rating Scale; CERAD, Consortium to Establish a Registry for Alzheimer’s Disease; Cho, choline; CSF, cerebrospinal fluid; EOAD, early-onset Alzheimer’s disease; EAD, early-onset Alzheimer’s disease; EOAD, autosomal dominant early−onset Alzheimer’s disease; ERP, Event Related Potentials; FA, fractional anisotropy; FAD, familial Alzheimer’s disease; fMRI, functional magnetic resonance imaging; Glx, glutamate/glutamine; HA, hippocampal atrophy; ICHD-2, International Classification of Headache Disorders; MCs, mutation carriers; MetO, methionine sulfoxide; ml, myo-inositol; MMSE, Mini-Mental State Examination; MRI, magnetic resonance imaging; MSR, magnetic resonance spectroscopy; MVP, memory verbal prose; NAA, N-acetyl-aspartate + N-acetyl-aspartyl-glutamate; NCs, non-carriers; OC, anti-fibrillar oligomer; OCTA, optical coherence tomography angiography; PCR, polymerase chain reaction; RFLP, restriction fragment length polymorphism analysis; WAIS, The Wechsler Adult Intelligence Scale; WCST, Wisconsin Card Sorting; WM, white matter; and αAPF, anti-annular protofibril.*

#### *PSEN1* and *APP*

Six of the studies included genetic analysis in combination with biomarkers (Ringman et al., 2008b, 2012a,b,c,d,e): four CSF quantifications, two of which identified Aβ42 depletion (Ringman et al., 2008b, 2012e), one finds oligomers elevation (Ringman et al., 2012d), and one identified overlapped protein changes (Ringman et al., 2012a). Three articles measured plasma levels; one found elevated Aβ42 (Ringman et al., 2008b), other inflammatory markers (Ringman et al., 2012b), and other elevated MetO (Ringman et al., 2012c).

In imaging studies (Ringman et al., 2007b,^2010^, ^2011^; Apostolova et al., 2011; Braskie et al., 2012, 2013; Lee et al., 2013; Joe et al., 2019; Singer et al., 2021), Ringman et al. (2007b) reported a decreased FA in white matter in the preclinical stage. Singer et al. (2021) found increased retinal perfusion also in presymptomatic carriers, and Joe et al. (2019) revealed that carriers had significantly lower levels of NAA and glutamine in the left pregenual anterior cingulate cortex, and lower levels of NAA and higher levels of myoinositol and choline in the precuneus, and a thinning of the posterior association and frontal cortices with hippocampal atrophy (Apostolova et al., 2011), lower thalamic, caudate, putamen volumes (Lee et al., 2013), and decreased BOLD activation in the anterior cingulate gyrus (Ringman et al., 2011). One article examined the ability of radiologists in the diagnosis of early AD stages in people in early stages of EOAD and found it to be suboptimal. Therefore, another marker is considered necessary for diagnosis (Ringman et al., 2010). Braskie et al. (2012) identified mutation carriers showing an increased fMRI activity in the fusiform and middle temporal gyri and a greater retrieval period signal, including in the frontal and temporal lobes (Braskie et al., 2013). These findings were related to the predementia phase.

Three of the studies with neuropsychological findings (Ringman et al., 2011; Braskie et al., 2012; Lee et al., 2013; Medina et al., 2021) reported no differences in cognitive tests between preclinical carriers of the mutations and non-carriers (Ringman et al., 2011; Braskie et al., 2012; Lee et al., 2013). However, in a memory retrieval task, a lower fMRI activity in the hippocampus was observed (Braskie et al., 2012) while for the executive function, the response gets slower as they approach the age onset of dementia (Medina et al., 2021). In carriers with mild cognitive impairment, lower memory, language, and visuospatial, executive functioning scores, were observed compared to preclinical carriers and controls (Lee et al., 2013).

In the clinical, a higher prevalence of headaches in MCs is held for different *PSEN1* and *APP* mutations (Ringman et al., 2008a) and electrophysiological features related to longer latencies of the N100, P200, N200, and P300 components, and smaller slow wave amplitudes (Golob et al., 2009).

#### *PSEN1*, *PSEN2*, and *APP*

Two of the studies analyzed brain tissue; Albrecht et al. (2009) identified that Casp-6 immunoreactivity was active in every participant, while Ringman et al. (2016) found Lewy body pathology in 27.1% of the ADAD cases and a higher Braak scores and cerebral amyloid angiopathy (CAA) prevalence. One study focused on linguistic aspects of the carriers (Medina et al., 2011), in which p-density was neither related to the status of participants with a history of EOAD nor with years to clinical onset of the disease, but it was associated with the presence of the *APOEe4* allele. The last article reported cognitive data in relation to neuroanatomical findings (Petok et al., 2018), in which more errors in generalization tasks were associated with smaller left hippocampal volume in carriers.

### History of Early-Onset Alzheimer’s Disease

Two studies were conducted on participants with a history of EOAD. One of the articles assessed cultural beliefs related to AD and genetic testing (Withers et al., 2019); in this study, some of the participants with a history of ADAD associated with genetic variants know their own risk of developing the disease. The authors found that providing information about the genetic bases of AD increased the interest of people with a history of ADAD for genetic testing. The other article assessed the experiences and needs of caregivers (Withers et al., 2021) and found the stress of informal caregivers comes from different sources beyond caregiving, such as knowing their own risk, caring for other family members including children, providing financially, their own health, and diagnosis access, and their main need is to access information in their own language.

## Discussion

The most common cause of hereditary EOAD is *PSEN1* mutations followed by *PSEN2* and *APP* mutations (Ramos et al., 2020). While the pathophysiology is similar, there are differences in the AD phenotype (Ringman et al., 2014). Among the variants in *PSEN1*, A431E is one of the primary three due to the number of carriers, which varies from 381 to 301 (Dumois-Petersen et al., 2020; Llibre-Guerra et al., 2021), and more descendants have been reported to be at risk. Therefore, the incidence is currently open (Llibre-Guerra et al., 2021).

This scoping review aims to synthesize the findings related to the characteristics of the *PSEN1* A431E variant associated with EOAD. The results of this review integrated a few studies focused only on the variant of our interest; most studies included other *PSEN1, PSEN2*, and *APP* variants in addition to A431E.

In Mexico, a founder effect (Ala431Glu in *PSEN1*) was hypothesized in the Altos de Jalisco area by Yescas et al. (2006). This could explain why the carriers were mainly Mexican and Mexican Americans both in Mexico and in the United States.

The founder effect hypothesis and the identification of more families could be associated with the growing interest in the variant reflected in the considerable increase in studies between 2006 and 2010 and it has been rising since then.

Analyzing the pathophysiology of EOAD-associated variants, such as A431E, from the early stages of the disease, provides comprehensive knowledge especially to identify biomarkers and interventions with therapeutic potential (Pilotto et al., 2013; Russell et al., 2014). We consider this could be the reason why most of the identified studies have been performed at the preclinical stage.

Synthesizing the findings of the A431E variant is crucial, for it allows comparison of phenotypic features between this and other variants and with SAD cases as well, and provides a framework for clinicians working with individuals with such a history of AD. Therefore, we highlight the importance of constructing knowledge by means of independently reporting results or comparing variants in studies that involve people with a history of two or more variants.

### A431E in *PSEN*

The average age of dementia onset is 40 years with a range of either 34–48 years (Yescas et al., 2006) or 42.5 ± 3.9 years of age (Dumois-Petersen et al., 2020). In both studies, the onset of symptoms was established based on reports from family members. Since families usually seek medical attention in advanced stages, we consider these reports may be biased. There were no longitudinal studies among the articles included in this review, only one study reported cross-sectional data over the course of 6 years of disease evolution (Dumois-Petersen et al., 2020). This lack of longitudinal studies may be due to the relatively recent identification of A431E, barriers that delay diagnosis such as the cost and time implications of assessments, and the attitudes of people with a history of the variant toward genetic analysis and research.

Evidence regarding phenotypic variability is not conclusive. Joshi et al. (2012) identified atypical features in carriers of variants associated with FAD, and these features were also reported by other authors: Pseudobulbar effect was identified also in the case of Parker et al. (2019); myoclonus was presented in one of the families identified by Yescas et al. (2006); gait abnormality was identified as a first symptom (Dumois-Petersen et al., 2020); and headaches were reported in the case of an A431E carrier (Alakkas et al., 2020), but the prevalence and intensity of the headaches could not be inquired; in addition, headaches were present in 67% of carriers in the study by Ringman et al. (2008a) and allowed for differentiating carriers and non-carriers of variants in both *PSEN1* and *APP* in a sample composed mainly of families with a history of A431E. Could these symptoms be part of a continuum or manifest at any moment is a topic of study and could be addressed in upcoming longitudinal studies. Spastic paraparesis is one of the main clinical features associated with A431E (Parker et al., 2019; Dumois-Petersen et al., 2020; Santos-Mandujano et al., 2020). In addition, pure motor presentations have been frequently identified in carriers of this variant (Llibre-Guerra et al., 2021). A431E has been associated with white matter abnormalities which correlate with motor impairments (Santos-Mandujano et al., 2020) which in some cases even preceded and exceeded cognitive symptoms.

Yescas et al., 2006, reported that one of the families in their study had a history of partial seizures 20 years prior to the clinical onset of AD, which they did not consider to be related to the A431E phenotype. However, seizures have been reported to occur in 15% of A431E carriers (Dumois-Petersen et al., 2020). The small population in the Yescas study may have been responsible for that finding and EEG evaluation in the early stages of the disease or a specific questioning for epileptic activity may be considered in EOAD cases, especially in those harboring the A431E variant.

Although evidence is still inconclusive, this type of manifestation could indicate A431E leads to an atypical presentation of EOAD whose early manifestations in the preclinical stage are not amnestic; therefore, this symptomatology should be explored as part of the clinical practice in this disease.

Given the heterogeneous characteristics of the studies involving carriers and non-carriers of different variants in the three principal genes associated with EOAD, we considered discussing the findings of A431E, *PSEN1*, *PSEN2*, and *APP* in a unified manner to contrast characteristic types among carriers of different variants.

Neuropsychological reports are inconsistent. Although memory deficits have been reported as the first cognitive symptom (Dumois-Petersen et al., 2020), verbal memory test scores did not allow differentiation between carriers and non-carriers at the preclinical stage (Ringman et al., 2004, 2011). In language, findings varied between those in which performance on language tests allowed differentiation (Lee et al., 2013) or not (Ringman et al., 2005; Medina et al., 2011) between carriers and non-carriers, and language disorders were observed as an initial symptom only in a minority (Dumois-Petersen et al., 2020). Visuospatial deficits are present since the early stages of the disease (Ringman et al., 2005; Lee et al., 2013; Santos-Mandujano et al., 2020). As for executive functioning, in mild cognitive impairment, both the carriers (Lee et al., 2013) and non-demented (Ringman et al., 2005) had lower and slower performance (Medina et al., 2021). These changes have been associated with Tau neurofibrillary tangles in the prefrontal areas (Weintraub et al., 2012) and may reflect dual protein participation in the early stages of the disease.

Neuropsychiatric manifestations complicate the diagnosis and even disguise some of the symptoms of the clinical onset of EOAD, as in the case reported by Alakkas et al. (2020). Thus, we highlight the importance of exploring a family history of AD in the neuropsychiatric clinical practice and following up over time. Depressive symptoms are common during EOAD associated with A431E in 53% of the cases (Dumois-Petersen et al., 2020). In the case of female carriers, depressive symptoms usually appear in the first stages of the disease and could be associated with the neuropathology of AD (Ringman et al., 2004). Other neuropsychiatric symptoms were reported infrequently in the studies reviewed, including hallucinations in 11.8% of the A431E carriers (Dumois-Petersen et al., 2020), catatonia, mutism, lack of spontaneous movement, and refusal to eat (Alakkas et al., 2020).

The identified neuropathological and biomarker findings and distinctive clinical features, such as SP, support the classification of the A431E variant as pathogenic, as described by the ACMG/AMP guidelines (Alzforum, 2022).

A neuropathological characterization of a brain of an A431E carrier showed severe frontal atrophy, neuronal loss, and gliosis from moderate to severe, and a predominance of neurofibrillary tangles followed by cotton-wool plaques, with greater accumulation of the beta-amyloid 40. The type and concentration per area of amyloid deposits and neurofibrillary tangles have been reported to differ widely among carriers of *PSEN1* and *PSEN2* variants and SAD (Maarouf et al., 2008).

Lewy body pathology (LBP) was more common in the amygdala of carriers of variants in *PSEN1* (found in 96% of carriers) than in *PSEN2* (Leverenz et al., 2006). Ringman et al. (2016), when analyzing middle frontal, superior temporal, and inferior parietal regions, reported LBP in only 21.1% of the ADAD cases due to variants in *PSEN1*, *PSEN2*, and *APP*. In these regions, this pathology type was significantly higher in SAD.

In contrast, CAA scores were higher in ADAD due to variants in *PSEN1* beyond codon 200 (in 63.3% of the cases analyzed) than in SAD (39.2%) (Ringman et al., 2016). Cases with ADAD had a CAA mean score situated in the mild range with a trend toward the moderate (Ringman et al., 2016). Similarly, the case described by Maarouf et al. (2008) of A431E had a moderate CAA score.

Activation levels of mtUPR genes in the frontal cortex were significantly higher in *PSEN1* variant carriers (70–90%) compared with levels in those with SAD (40–60%) (Beck et al., 2016). This may result in increased vulnerability to pathological processes associated with this response in carriers of *PSEN1* variants.

Active Casp-6 immunoreactivity is present in cases of EOAD due to variants in *PSEN1*, *PSEN2*, and *APP*, and in SAD (Albrecht et al., 2009). The two A431E carriers in this study presented a neuritic plaque and neurofibrillary tangle in densities ranging from moderate to severe and mild to moderate neuropil threads in the superior/medial temporal gyrus, hippocampus, and entorhinal cortex (Albrecht et al., 2009).

Although the sample size of these studies was small and the minority were carriers of A431E, it is important to highlight that the neuropathological findings in postmortem studies allow in the first instance the differentiation between EOAD due to A431E variant or others in *PSEN1*, and variants in *PSEN2* or *APP*, SAD, and dementias caused by other conditions such as PPA and bvFTD (Gefen et al., 2020), and may ultimately be of help for the differential diagnosis (Braak and Braak, 1991; McKhann et al., 2011).

Case reports identified atrophy disproportionate to age (Parker et al., 2019; Alakkas et al., 2020; Santos-Mandujano et al., 2020) in structures such as thalamic, caudate, the putamen (Lee et al., 2013), hippocampus, and in posterior association and frontal cortices (Apostolova et al., 2011). As in the case of Alakkas et al. (2020), levels of cortical and subcortical atrophy are key in the differential diagnosis of the disease, but there are also other potential measures in the identification of EOAD, for example, decreased BOLD activation in the cingulate gyrus (Ringman et al., 2011), hyperactivity in the fusiform gyrus and medial temporal gyrus (Braskie et al., 2012), and lower levels of NAA and higher levels of myoinositol and choline in the precuneus (Joe et al., 2019).

Biomarkers are useful, especially CSF tau/ratio for differential diagnosis, therapeutic targets, and even to measure the progression of the disease (Pilotto et al., 2013; Russell et al., 2014). In CSF, toxic effects of Aβ42 oligomerization at synapses independent of amyloid plaque formation have been studied (Ringman et al., 2012d), and Ringman et al. (2012e) found elevated oligomers and low levels of Aβ42 in asymptomatic individuals with a history of variants in *PSEN1* (including A431E) and *APP*. Of the seven participants, five were carriers, with a significant elevation of ring protofibrils with a progressive reduction identified 20 years before to age of onset of dementia. In carriers, the Aβ42/Aβ40 ratio was lower (Ringman et al., 2008b) whereas SAD participants and A431E carriers had similar changes in Aβ42 and Aβ16, but symptomatic and asymptomatic carriers show a downward trending pattern of Aβ37, Aβ38, and Aβ39 isoform levels, suggesting that this variant determines the cleavage site of γ-secretase which is associated with disease manifestation (Portelius et al., 2010).

Plasma Aβ42 levels have been identified as elevated in carriers of variants associated with FAD and may decrease with disease progression prior to the development of dementia (Ringman et al., 2008b). An association was found between methionine sulfoxide levels with the amount of plasma F2-isoprostane and superoxide dismutase-1 (Ringman et al., 2012c); in addition, inflammatory markers and synaptic degeneration in presymptomatic carriers have been found, which has been indicated as a potential therapeutic target (Ringman et al., 2012b).

Providing information about the genetic basis of AD may increase the interest of people with a history of variants associated with EOAD to participate in clinical research and act focused on staying informed about the implications of this disease (Withers et al., 2019). However, it is important to consider that few studies have been conducted in this specific area. Other authors identified the factors leading to genetic testing in individuals with a history of variants associated with FAD were worry about the clinical onset of the disease and if they were carriers or not, and family and financial planning (Steinbart et al., 2001). The financial aspect was also an important stress source for caregivers of variant carriers associated with EOAD since some of them were also the main financial providers of their families (Withers et al., 2021).

An expansion of the findings regarding A431E is to be expected because a current area of research in our University is focused on studying the clinical, neuropsychological, social, and pathological hallmarks of these variants and has started collaborations with groups devoted to the ADAD. We expect that broader, longitudinal studies in both the preclinical and clinical stages will also help in a better understanding of the clinical course and the physiopathology of EOAD with the A431E variant.

### Limitations

The limitations in the methodology consist of having only considered studies in humans or brain tissue. The exclusion of experimental studies could result in relevant information on pathophysiology, which was omitted from this review.

This article has described the extent, type, and research findings related to A431E in *PSEN1*. However, quality criteria were not applied to assess the methods of the included studies or the validity of their results.

Although among many of the studies identified, participants were carriers of other variants of *PSEN1* or associated with FAD, and most of the participants carried the A431E represents a limitation when characterizing these carriers. We recommend avoiding the generalization of the different findings.

In addition, the number of participants in each study and the design are variable.

Future systematic reviews should integrate more information about other variants mostly studied to contrast the information found.

## Conclusion

This scoping review summarizes research associated with the A431E variant of *PSEN1* associated with EOAD.

In total, we reviewed 42 studies, all of them cross-sectional, seven focused on A431E, 11 analyzed that variant and others in the *PSEN1* and *PSEN2* genes, and 24 whose samples are composed of cases in the genes *PSEN* and *APP.*

The included studies indicate that A431E has been studied in several categories, such as genetics, clinical, imaging, neuropsychology, neuropathology, and biomarkers in carriers or participants with a history of the variant in the preclinical and clinical phases.

The key findings in these studies identify several changes that occur years before the age of onset of dementia. Further studies can be designed to monitor through time the early changes associated with the disease, allowing the establishment of programs to attend to the needs of these families.

The clinical heterogeneity found in the different studies is diagnosis challenging, which in clinical practice represents a burden for health professionals and public health measures, where families are affected by late diagnosis, delaying their intervention and support.

This study provides a helpful synthesis for researchers and clinicians who work with AD-related gene variants carriers, mostly with early-onset familial AD.

## Data Availability Statement

The original contributions presented in the study are included in the article/Supplementary Material, further inquiries can be directed to the corresponding author.

## Author Contributions

MO-B made the conception and study design. MO-B, YO-R, AC-A, and VS-G performed the database search. MO-B and YO-R performed data analysis. MO-B, YO-R, VS-G, and AC-A wrote the manuscript screening. All authors contributed with feedback and edited the manuscript.

## Conflict of Interest

The authors declare that the research was conducted in the absence of any commercial or financial relationships that could be construed as a potential conflict of interest.

## Publisher’s Note

All claims expressed in this article are solely those of the authors and do not necessarily represent those of their affiliated organizations, or those of the publisher, the editors and the reviewers. Any product that may be evaluated in this article, or claim that may be made by its manufacturer, is not guaranteed or endorsed by the publisher.
