# Supplementary material for: PSEN1 c.1292C<A Variant and Early-Onset Alzheimer’s Disease: A Scoping Review
Source: Front Aging Neurosci. 2022 Jul 22;14:860529. doi: 10.3389/fnagi.2022.860529 (PMC9361039; doi:10.3389/fnagi.2022.860529)
Supplement: Supplementary file 1 [file Table_1.DOCX]

Supplementary Material

# Supplementary Figures and Tables

**Table S1.** General details of the study

| **Ref.** | **Tittle** | **Country** | **Study group** | **Sample** |
| --- | --- | --- | --- | --- |
| Rogaeva et al., 2001 | Screening for PS1 mutations in a referral-based series of AD cases: 21 novel mutations | USA, Germany and Canada | Subjects with a diagnosis of AD, symptomatic and asymptomatic, of which the majority (78%) of the cases were of early onset associated with variants in PSEN1 | Humans |
| Ringman et al., 2004 | Female preclinical presenilin-1 mutation carriers unaware of their genetic status have higher levels of depression than their non-mutation carrying kin | USA | Women at risk of inheriting PSEN1 variants (A431E and L235V) | Humans |
| Ringman et al., 2005 | Neuropsychological function in nondemented carriers of presenilin-1 mutations | USA | Subjects at risk of inheriting PSEN1 variants (A431E and L235V) | Humans |
| Yescas et al., 2006 | Founder effect for the Ala431Glu mutation of the presenilin 1 gene causing early-onset Alzheimer's disease in Mexican families | Mexico | Families at risk of familial AD due to PSEN1 variants | Humans |
| Murrell et al., 2006 | The A431E mutation in PSEN1 causing Familial Alzheimer’s Disease originating in Jalisco State, Mexico: an additional fifteen families | USA | Subjects at risk of inheriting A431E variant | Humans |
| Leverenz et al., 2006 | Lewy Body Pathology in Familial Alzheimer Disease: Evidence for Disease- and Mutation-Specific Pathologic Phenotype | USA | Brain tissue from PSEN1 and PSEN2 mutation carriers | Brain tissue |
| Ringman et al., 2007a | Performance on MMSE sub-items and education level in presenilin-1 mutation carriers without dementia | Mexico | Subjects at risk of inheriting PSEN1 variants | Humans |
| Ringman et al., 2007b | Diffusion tensor imaging in preclinical and presymptomatic carriers of familial Alzheimer's disease mutations | USA | PSEN1 or APP mutation carriers and noncarriers | Humans |
| Ringman et al., 2008a | Increased prevalence of significant recurrent headache in preclinical familial Alzheimer's disease mutation carriers | Mexico and USA | Subjects at risk of inheriting PSEN1 or APP variants | Humans |
| Ringman et al., 2008b | Biochemical markers in persons with preclinical familial Alzheimer disease | USA | Subjects at risk of inheriting PSEN1 or APP variants | Humans |
| Maarouf et al., 2008 | Histopathological and molecular heterogeneity among individuals with dementia associated with Presenilin mutations | USA | Brain tissue from carriers of PSEN1 and PSEN2 variants, from people with sporadic AD and healthy controls | Brain tissue |
| Golob et al., 2009 | Cortical event-related potentials in preclinical familial Alzheimer disease | USA | PSEN1 or APP mutation carriers and noncarriers | Humans |
| Albrecht et al., 2009 | Caspase-6 Activation in Familial Alzheimer Disease Brains Carrying Amyloid Precursor Protein, Presenilin I or Presenilin II Mutations | USA | PSEN1, PSEN2 and APP mutation carriers | Brain tissue |
| Ringman et al., 2010 | Insensitivity of visual assessment of hippocampal atrophy in familial Alzheimer’s disease | USA | Subjects at risk of inheriting PSEN1 or APP variants | Humans |
| Portelius et al., 2010 | Distinct cerebrospinal fluid amyloid β peptide signatures in sporadic and PSEN1 A431E-associated familial Alzheimer's disease | Sweden | A431E carriers, subjects with sporadic AD, subjects with depression and healthy controls | Humans (cerebrospinal fluid) |
| Ringman et al., 2011 | Effects of Risk Genes on BOLD Activation in Presymptomatic Carriers of Familial Alzheimer's Disease Mutations during a Novelty Encoding Task | USA | Subjects at risk of inheriting PSEN1 or APP variants | Humans |
| Medina et al., 2011 | Propositional Density and Apolipoprotein E Genotype among Persons at Risk for Familial Alzheimer's Disease | USA | Subjects with variants associated with familial AD | Humans |
| Apostolova et al., 2011 | Cortical and Hippocampal Atrophy in Patients with Autosomal Dominant Familial Alzheimer's Disease | USA | Subjects at risk of inheriting PSEN1 or APP variants | Humans |
| Ringman, 2012a | Proteomic Changes in Cerebrospinal Fluid of Presymptomatic and Affected Persons Carrying Familial Alzheimer Disease Mutations | USA | Carriers and subjects at risk of inheriting PSEN1 or APP variants | Humans (cerebrospinal fluid) |
| Ringman et al., 2012b | Plasma Signaling Proteins in Persons at Genetic Risk for Alzheimer Disease: Influence of APOE Genotype | USA | Carriers and subjects at risk of inheriting PSEN1 or APP variants | Humans |
| Ringman et al., 2012c | Plasma methionine sulfoxide in persons with familial Alzheimer’s disease mutations | USA | Subjects at risk of inheriting PSEN1 or APP variants | Humans |
| Ringman et al., 2012d | Conformation-dependent oligomers in cerebrospinal fluid of presymptomatic familial Alzheimer's disease mutation carriers | USA | Subjects at risk of inheriting PSEN1 or APP variants | Humans (cerebrospinal fluid) |
| Ringman et al., 2012e | Cerebrospinal Fluid Biomarkers and Proximity to Diagnosis in Preclinical Familial Alzheimer's Disease | USA | Subjects at risk of inheriting PSEN1 or APP variants | Humans |
| Joshi et al., 2012 | Comparison of clinical characteristics between familial and non-familial early onset Alzheimer’s disease | USA | Subjects with sporadic AD and familial AD | Humans |
| Braskie et al., 2012 | Increased fMRI signal with age in familial Alzheimer’s disease mutation carriers | USA | PSEN1 or APP mutation carriers and noncarriers | Humans |
| Braskie et al., 2013 | Memory performance and fMRI signal in presymptomatic familial Alzheimer's disease | USA | Subjects at risk of inheriting PSEN1 or APP variants | Humans |
| Roher et al., 2013 | Subjects harboring presenilin familial Alzheimer’s disease mutations exhibit diverse white matter biochemistry alterations | USA | PSEN1 or PSEN2 mutation carriers | Brain tissue |
| Lee et al., 2013 | Regional brain volume differences in symptomatic and presymptomatic carriers of familial Alzheimer’s disease mutations | USA | PSEN1 or APP mutation carriers and noncarriers | Humans |
| Soosman et al., 2016 | Widespread white matter and conduction defects in PSEN1-related spastic paraparesis | USA | PSEN1 mutation carriers | Humans |
| Ringman et al., 2016 | Neuropathology of Autosomal Dominant Alzheimer Disease in the National Alzheimer Coordinating Center Database | USA | Carriers of PSEN1, APP and PSEN 2 and sporadic AD | Humans |
| Beck et al., 2016 | Evidence for Mitochondrial UPR Gene Activation in Familial and Sporadic Alzheimer’s Disease | USA | Frontal cortex brain tissue samples from cases of sporadic AD, familial AD (associated with PSEN 1) and healthy controls | Brain tissue |
| Petok et al., 2018 | Impairment of memory generalization in preclinical autosomal dominant Alzheimer’s disease mutation carriers | USA | PSEN1, PSEN2 or APP mutation carriers and noncarriers | Humans |
| Withers et al., 2019 | A mixed-methods study of cultural beliefs about dementia and genetic testing among Mexicans and Mexican-Americans at-risk for autosomal dominant Alzheimer’s disease | Mexico and USA | Subjects at risk of inheriting familial AD | Humans |
| Parker et al., 2019 | Homozygosity for the A431E mutation in PSEN1 presenting with a relatively aggressive phenotype | USA | A431E mutation carriers | Humans |
| Joe et al., 2019 | H MRS Spectroscopy in Preclinical Autosomal Dominant Alzheimer Disease | USA | Subjects at risk of inheriting PSEN1, PSEN2, or APP variants | Humans |
| Santos-Mandujano et al., 2020 | Clinical Association of White Matter Hyperintensities Localization in a Mexican Family with Spastic Paraparesis Carrying the PSEN1 A431E Mutation | Mexico | A431E mutation carriers | Humans |
| Dumois‐Petersen et al., 2020 | Autosomal dominant early onset Alzheimer's disease in the Mexican state of Jalisco: High frequency of the mutation PSEN1 c.1292C>A and phenotypic profile of patients | Mexico | Subjects at risk of inheriting A431E PSEN1 mutation | Humans |
| Gefen et al., 2020 | Primary Progressive Aphasia has a Unique Signature Distinct from Dementia of the Alzheimer’s Type and Behavioral Variant Frontotemporal Dementia Regardless of Pathology | USA | Cases with ante-mortem diagnosis of progressive primary aphasia, behavioral variant of frontotemporal dementia and Alzheimer's dementia | Brain tissue |
| Alakkas et al., 2020 | Early-Onset Alzheimer's Disease Masquerading as Catatonia | USA | Variant carrier woman in PSEN1 | Humans |
| Medina et al., 2021 | Reaction time and response inhibition in autosomal dominant Alzheimer's disease | USA | Subjects at risk of inheriting variants associated with familial AD | Humans |
| Withers et al., 2021 | “My backpack is so heavy”: Experiences of Latino caregivers of family with early-onset Alzheimer's | USA and Latinos | Caregivers of people with early-onset AD | Humans |
| Singer et al., 2021 | Abnormal retinal capillary blood flow in autosomal dominant Alzheimer's disease | USA | PSEN1 or APP mutation carriers and noncarriers | Humans (retinal histopathology) |

AD: Alzheimer disease, APOE: apolipoprotein E, BOLD: blood oxygen level—dependent, fMRI: functional magnetic resonance imaging, MMSE: Mini-mental State Examination. MRS: magnetic resonance spectroscopy, and UPR: unfolded protein response.
